# Preclinical study of using multiphoton microscopy to diagnose liver cancer and differentiate benign and malignant liver lesions (Erratum)

**DOI:** 10.1117/1.JBO.31.9.099801

**Published:** 2026-09-26

**Authors:** Jun Yan, Shuangmu Zhuo, Gang Chen, Xiufeng Wu, Dong Zhou, Shusen Xie, Jiahao Jiang, Mingang Ying, Fan Jia, Jianxin Chen, Jian Zhou

**Affiliations:** a Fudan University, Zhongshan Hospital, Liver Cancer Institute, Shanghai, China; b Fujian Normal University, Institute of Laser and Optoelectronics Technology, Fujian Provincial Key Laboratory for Photonics Technology, Key Laboratory of Optoelectronic Science and Technology for Medicine of Ministry of Education, Fuzhou, China; c Teaching Hospital of Fujian Medical University, Fujian Provincial Tumor Hospital, Department of Surgery, Fuzhou, China; d Teaching Hospital of Fujian Medical University, Fujian Provincial Tumor Hospital, Department of Pathology, Fuzhou, China

## Abstract

The erratum corrects panel B in Figure 1.

This article [*J. Biomed. Opt.* **17**(2), 026004 (2012) [DOI: 10.1117/1.JBO.17.2.026004] was originally published on 22 February 2012 with an incorrect image inadvertently inserted into Fig. 1(b). The authors sincerely apologize for this error and any confusion it may have caused for readers.

The original and corrected images are provided here.

• Original:

**Figure:**
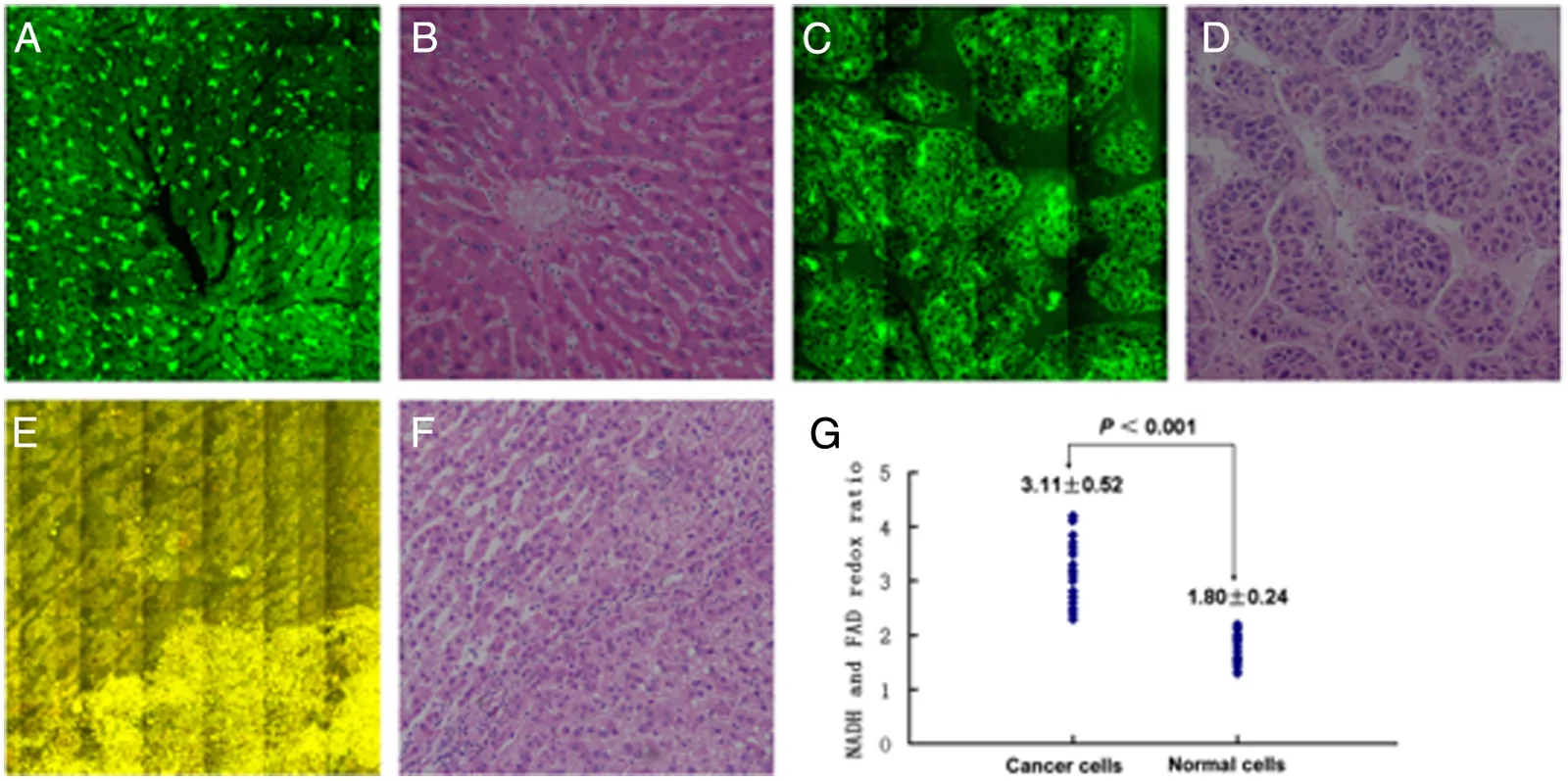


• Corrected:

**Figure:**
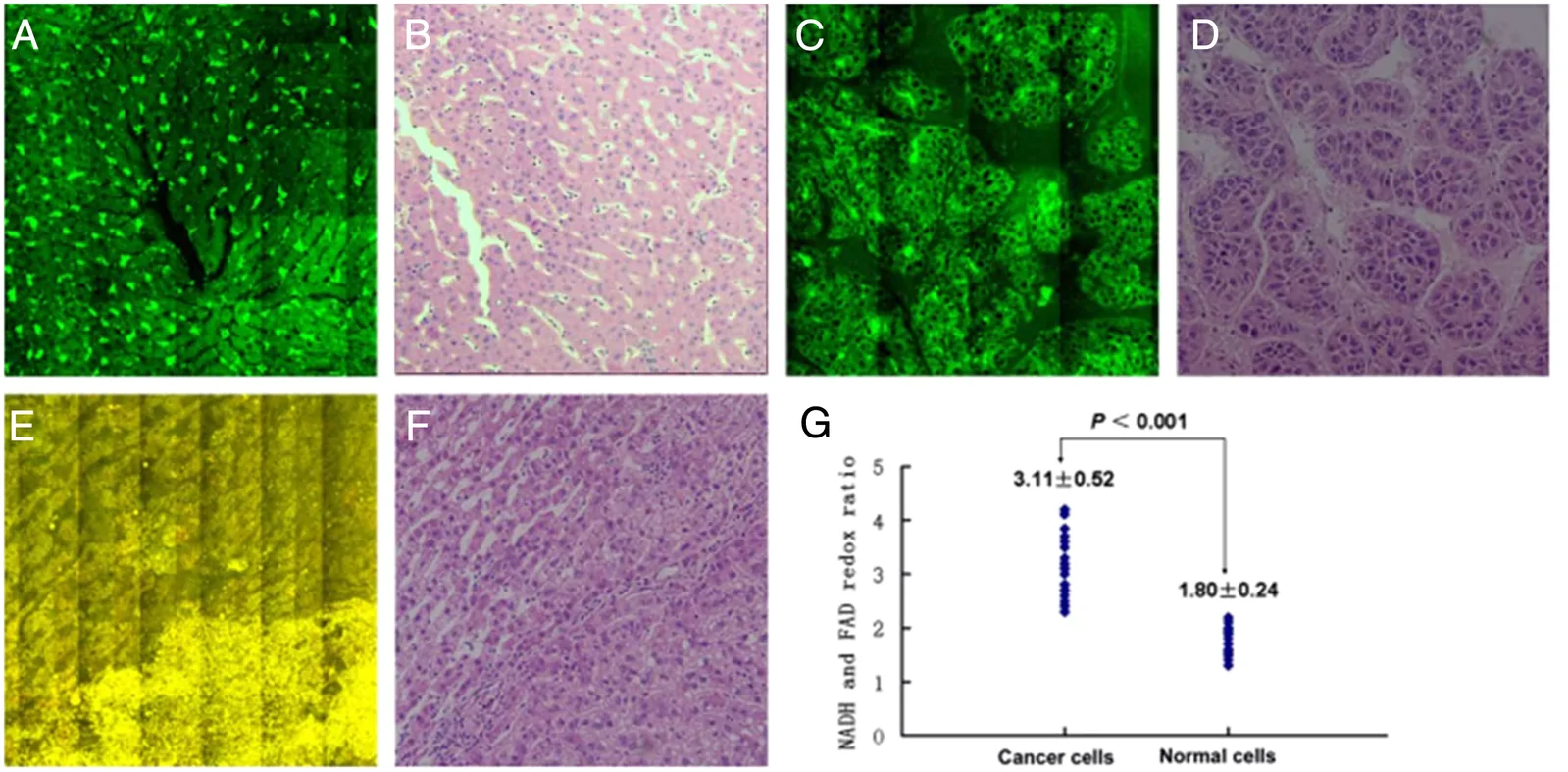


The figure caption was correct in the original and remains unchanged. The article was corrected and republished 10 September 2026.

All co-authors have read, reviewed, and endorsed this correction notice. Importantly, this amendment does not affect or change any of the scientific conclusions drawn in the original article.

